# Effects of conservation management of landscapes and vertebrate communities on Lyme borreliosis risk in the United Kingdom

**DOI:** 10.1098/rstb.2016.0123

**Published:** 2017-04-24

**Authors:** Caroline Millins, Lucy Gilbert, Jolyon Medlock, Kayleigh Hansford, Des BA Thompson, Roman Biek

**Affiliations:** 1 Institute of Biodiversity, Animal Health and Comparative Medicine, University of Glasgow, Glasgow G12 8QQ, UK; 2 The Boyd Orr Centre for Population and Ecosystem Health, University of Glasgow, Glasgow G12 8QQ, UK; 3 School of Veterinary Medicine, University of Glasgow, Glasgow G61 1QH, UK; 4 The James Hutton Institute, Craigiebuckler, Aberdeen AB15 8QH, UK; 5 Medical Entomology Group, Emergency Response Department, Public Health England, Salisbury, SP4 0JG, UK; 6 Health Protection Research Unit in Environment and Health, Porton Down, Salisbury SP4 0JG, UK; 7 Scottish Natural Heritage, 231 Corstorphine Road, Edinburgh, EH12 7AT, UK

**Keywords:** Lyme borreliosis, *Ixodes*, conservation management, biodiversity

## Abstract

Landscape change and altered host abundance are major drivers of zoonotic pathogen emergence. Conservation and biodiversity management of landscapes and vertebrate communities can have secondary effects on vector-borne pathogen transmission that are important to assess. Here we review the potential implications of these activities on the risk of Lyme borreliosis in the United Kingdom. Conservation management activities include woodland expansion, management and restoration, deer management, urban greening and the release and culling of non-native species. Available evidence suggests that increasing woodland extent, implementing biodiversity policies that encourage ecotonal habitat and urban greening can increase the risk of Lyme borreliosis by increasing suitable habitat for hosts and the tick vectors. However, this can depend on whether deer population management is carried out as part of these conservation activities. Exclusion fencing or culling deer to low densities can decrease tick abundance and Lyme borreliosis risk. As management actions often constitute large-scale perturbation experiments, these hold great potential to understand underlying drivers of tick and pathogen dynamics. We recommend integrating monitoring of ticks and the risk of tick-borne pathogens with conservation management activities. This would help fill knowledge gaps and the production of best practice guidelines to reduce risks.

This article is part of the themed issue ‘Conservation, biodiversity and infectious disease: scientific evidence and policy implications’.

## 1. Introduction

The management of landscapes and habitats for conservation is often driven by policies aiming to enhance biodiversity, to improve ecosystem services or to manage invasive species. These policy-driven land management changes include native woodland regeneration and restoration to optimize biodiversity, vegetation management, urban greening and the management of invasive or pest species. However, there may be unintended consequences of these management actions, such as effects on infectious-disease risk due to changes in wild vertebrate and vector population distribution, abundance and movement patterns [1,2]. As most significant human and livestock pathogens can infect many host species, changes in the host community composition can affect the persistence and prevalence of these pathogens [3–6]. This is because species within a host community contribute differently to pathogen dynamics due to differences in their abundance, infection prevalence and infectiousness [7].

In recent decades, there has been a global increase in the incidence of many emerging and endemic vector-borne diseases in humans [8,9]. Although some important vector-borne diseases such as malaria and dengue fever are maintained in a human-vector cycle, most vector-borne zoonotic pathogens are maintained by vectors and multiple wild vertebrate hosts, with humans acting as dead-end hosts [10]. Shifts in land use, alterations to host populations and climate change have been identified as the main drivers of endemic vector-borne pathogen emergence, which can result in invasion of the vector-borne pathogen into new areas, or cause increased intensity of transmission within enzootic areas [1,8,11]. Therefore, conservation management activities that alter the land use or control populations of invasive or pest species are likely to result in changes to the distribution and transmission dynamics of vector-borne pathogens. This is due to effects on vertebrate host communities, which provide blood meals for vectors, and effects on the abiotic conditions for vector development and off-host survival. For example, increased deer populations in Europe have been linked to rising numbers of *Ixodes ricinus* ticks, which transmit a number of pathogens important for human health and livestock [11]. Management of deer populations in areas where they are highly abundant is often necessary as part of woodland regeneration and biodiversity projects, with consequences for ecological communities, tick populations and pathogen transmission [12,13] (see §2).

Lyme borreliosis is among the most important vector-borne zoonoses in the Northern hemisphere and has increased in distribution and incidence across large parts of North America and the higher altitudes and latitudes of Europe, including the United Kingdom [14,15]. Transmitted by Ixodid ticks, the causative agents of Lyme borreliosis are spirochaete bacteria belonging to the *Borrelia burgdorferi sensu lato* species complex. There are at least 19 genospecies of *B. burgdorferi* s.l. in this group, some of which are pathogenic to humans [16,17]. In Western Europe, *I. ricinus* is the primary vector and is widely distributed across a range of ecoregions and habitats, and feeds on many species of birds, mammals and reptiles [18]. In the United Kingdom, four genospecies of *B. burgdorferi* s.l. have been detected in questing *I. ricinus* ticks [19–21]. These are small-mammal–associated *B. afzelii*, bird-associated *B. garinii*, *B. valaisiana* and the generalist genospecies *B. burgdorferi sensu* *stricto* which can be transmitted by Ixodid vectors feeding on competent reservoir hosts such as birds and small mammals [22–26]. The main route of transmission of *B. burgdorferi* s.l. is considered to be via transtadial transmission by ticks feeding on infected hosts that maintain infection through to the next life stage. Co-feeding and transovarial transmission can contribute to transmission in some circumstances [27–29]. The environmental risk to humans from Lyme borreliosis is defined as the density of infected tick vectors in the environment. The density of infected nymphs is usually focused on as the most abundant tick life-stage carrying the pathogen and is referred to as Lyme borreliosis risk throughout the rest of this paper. The probability of human exposure to these infected ticks will depend on human behaviour and how people interact with the environment. Therefore, awareness of tick-borne disease risk as well as mitigation strategies such as animal and habitat management can help to reduce human exposure to ticks.

**Figure 1.**
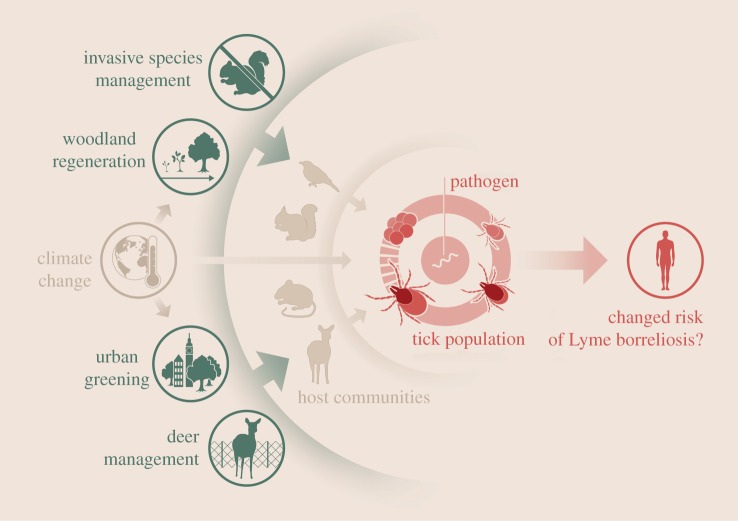
Overview figure of selected conservation management activities including invasive species management, woodland regeneration, urban greening and deer management that can affect vertebrate host communities, tick populations, pathogen transmission and the risk of Lyme borreliosis (© Diogo Guerra).

Here, we consider the effect of common conservation management practices on the environmental risk of Lyme borreliosis in the UK. We identify four relevant areas of conservation activities: deer management, woodland management and regeneration, control of invasive species, and urban greening (figure 1). We review the evidence that these management activities have on vector populations, host communities, pathogen transmission and the environmental risk of *B. burgdorferi* s.l. and discuss knowledge gaps and policy implications (table 1). While we focus on data and examples from the UK and continental Europe, the ecological mechanisms we discuss are likely to be relevant to any geographical area affected by Lyme borreliosis.

**Table 1.** Summary of the potential effects of different conservation management actions on vertebrate communities, *Ixodes ricinus* abundance and the risk of Lyme borreliosis.

type of conservation management	effect on vertebrate community?	effect on tick abundance?	effect on risk of Lyme borreliosis? (density of infected nymphs)
deer management (fencing or culling)	deer management can result in reduced deer abundance. Subsequent increases in vegetation from reduced browsing by deer can result in increased densities of competent small mammal hosts [12,30]	tick abundance would be expected to be reduced in the absence of alternate hosts for adult ticks [12]	increases in competent small mammal hosts may lead to an increased prevalence of infection, but reduced deer will reduce overall tick density, therefore the density of infected ticks (risk) will likely fall (assuming there are few alternative hosts for adult ticks)
woodland regeneration	an increase in populations of small mammals and birds (competent hosts) is predicted based on habitat-specific densities [31–33]. Deer populations (incompetent hosts) may increase if not controlled or excluded by fencing [12,34]	if deer are controlled to aid tree growth, ticks may be reduced or, if not, ticks may increase due to more favourable abiotic conditions for tick survival	the prevalence of infection is likely to increase, but the density of infected ticks may be increased or decreased depending on whether deer management is carried out
invasive species management	culling of invasive grey squirrels can result in decreased populations of this host. Populations of red squirrels may increase where present [35], populations of other competent hosts such as small mammals and birds may also increase in response	invasive species management is likely to have a limited effect in areas with tick reproduction hosts (e.g. deer)	unknown, but may lead to a decreased risk in the short term. Longer-term changes are dependent on the response of other competent small-mammal and bird populations to the removal of grey squirrels
urban greening	increased urban greenspace and connectivity will increase the abundance of both competent and incompetent vertebrate hosts in urban environments	a range of vertebrates support ticks and if larger animals (e.g. deer) are able to access urban greenspace then ticks can establish. The role of cats and dogs as tick hosts should be investigated [36]	although tick abundance may be lower in urban greenspace compared to rural areas, there is evidence that pathogen prevalence may be higher in those ticks (given that there is likely to be less of a dilution effect from large mammals) [37]. Also human exposure is likely to be higher in urban areas

## 2. Deer management

**Figure 2.**
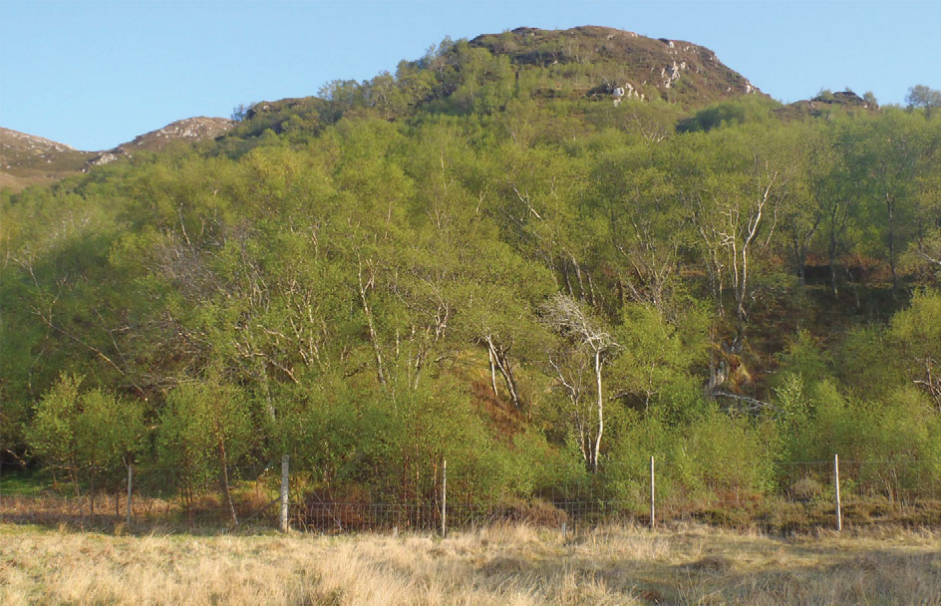
Woodland regeneration projects often incorporate exclusion fences for deer to reduce browsing (© Caroline Millins).

Conservation objectives often require the close management of large herbivores in order to improve habitat quality. Prime examples include woodland regeneration and improvement projects for biodiversity enhancement, which require management of deer to avoid damage of young trees and vegetation. Reducing grazing or browsing by deer can be achieved by exclusion fencing or culling [12], figure 2. As deer are important hosts of Ixodid ticks in many areas, changes in deer density can affect tick abundance with implications for the transmission of tick-borne pathogens [12,38].

Deer can feed large numbers of adult female ticks, which then lay eggs and produce the next generation of immature ticks, and deer are thus termed ‘tick reproduction hosts’ [39]. A great many studies have shown that deer can be instrumental in maintaining tick populations, such that areas with more deer also have more ticks [12,19,40–51] although there is some uncertainty in the precise relationship between deer density and tick density [52]. Some of these studies specifically tested the impact of deer management methods and, when deer numbers were reduced through culling or fencing, there were dramatic declines in the tick population. For example, a study examining *I. ricinus* tick density in response to deer management methods reported reductions of 73% on heather moorland and 94% in woodlands due to culling, and 86–88% due to fencing moorlands and 96% reductions due to fencing woodlands [12].

However, as deer are considered incompetent reservoir hosts for *B. burgdorferi* s.l. and do not infect feeding ticks [53–55], but see [56,57], increasing deer densities might not necessarily result in a higher risk of Lyme borreliosis. Mathematical models of tick-borne pathogens have predicted a non-linear relationship between deer density and the prevalence of tick-borne pathogens [58–60]. According to these models, initial increases in deer density cause increased pathogen prevalence, because more adult ticks feed successfully. Ticks in their early life stages tend to feed preferentially on small hosts that are often competent to transmit the pathogen. However, at very high deer densities, a reduction in prevalence is predicted because more and more immature ticks feed on deer that are not competent to transmit, known as a ‘dilution effect’ [38,61]. A dilution effect is defined as occurring when the addition of one or more host species to a community reduces the prevalence of a pathogen and decreases the likelihood of pathogen persistence [62].

Consistent with these predictions, a large empirical study in Italy found an increase in the prevalence of *B. burgdorferi* s.l. in ticks with increasing deer density up to a threshold of 15 deer/100 hectares (ha), after which prevalence decreased [43]. Due to positive effects of deer density on tick density, the risk of Lyme borreliosis (density of infected nymphs) continued to increase up to 60 deer/100 ha before decreasing slightly [43]. Other empirical studies have reported variable associations between deer density and *B. burgdorferi* s.l. prevalence, from positive [19] to negative [41,63] or neutral [51,64–66]. These inconsistent effects might be due to sampling that usually only covers part of the range of deer and competent reservoir host densities, which limits the chance of detecting non-linear relationships, as well as local variation in climatic factors affecting vector populations and host–vector interactions. Despite the variable effects of deer density on *B. burgdorferi* s.l. prevalence, it remains possible that deer density may be more consistently linked to the risk of Lyme borreliosis, apart from at exceptionally high deer densities when a dilution effect may occur. Some studies have reported positive effects of deer density on Lyme borreliosis risk [43,51,67], while other studies have found no significant effect [65,66,68]. Reported differences among studies probably relate to variation in the density of competent reservoir hosts between studies.

While reducing deer densities by fencing or culling will almost certainly result in dramatically decreased tick populations when there are no suitable alternative hosts, and may decrease the risk of Lyme, there are several important issues concerning both fencing and intensive culling. These include expense, ethics, public opinion and conflicting land management objectives. For example roe deer (*Capreolus capreolus*) are increasingly present in urban green space and the peri-urban fringe and act as important tick hosts. Public opinion and practical concerns may make it extremely difficult to manage urban deer populations by culling or fencing. Furthermore, deer move within urban areas by moving along green corridors, which can include peri-domestic habitats such as gardens. Habituation of urban deer populations to humans and attractive feeding areas within these areas can increase tick densities close to human dwellings and the risk of human exposure to ticks. Culling of deer to reduce the population density is only likely to be effective when conducted at the landscape scale, which may require cooperation between private and public land managers [69]. As deer are iconic animals with cultural value for tourism and hunting, intensive culls are not always desirable [12].

Managing deer populations where alternate hosts are present is likely to be less successful in reducing tick numbers. Livestock can act as hosts for all life stages of *I. ricinus*, and other species such as mountain hares (*Lepus timidus*) can maintain *I. ricinus* populations by feeding all three tick life-stages in the absence of larger vertebrates [27,70–72]. It may be difficult to maintain fenced exclosures to prevent all deer from entering, and may not be feasible to fence large areas. Fencing can be unsightly and unpopular with countryside users and can pose risks to birds of conservation importance such as capercaillie (*Tetrao urogallus*) and black grouse (*Tetrao tetrix*) [73].

There is also evidence that fencing can lead to shifts in host communities, as seen with mountain hare (*Lepus timidus*) and small mammal densities increasing on the inside of fenced areas [12,30]. Such increases in competent host densities in response to changes in vegetation could, in principle, result in a higher prevalence of *B. burgdorferi* s.l. inside fenced areas. Studies that monitor changes in small mammal communities and tick infestations as well as questing tick abundance and *B. burgdorferi* s.l. prevalence in response to deer density control are therefore needed to assess the effect of fencing on Lyme borreliosis risk.

## 3. Woodland regeneration and management

Many parts of Europe are currently experiencing major land use change due to woodland expansion. For Europe as a whole, there was an increase in forested land cover of more than 600 000 km^2^ between 1993 and 2006 [74]. While some woodland expansion is unintended due to reduced pasture management, increasing woodland cover is currently being encouraged under international and national policies [74–77]. Implementation of changes to land services is through existing policies including the European Union (EU) Biodiversity Action Plan, Article 10 of the Habitats Directive and the Strategic Environmental Assessment and Environmental Impact Assessment Directives [74]. Though, as the UK voted to leave the EU in 2016, EU policies may soon not apply.

The primary aim of these policies is to improve ecosystem services for climate change mitigation and to enhance biodiversity, water quality and human well-being. In the UK, forested land cover is currently much reduced from historic levels and is low in comparison with other countries globally and across Europe [75,78]. Government policy in Scotland is to increase woodland cover from 18% to 25% by 2050, requiring the creation of 10–15 000 hectares of woodland a year, while in England a target has been set to increase woodland cover from 9% to 12% by 2060 [76,78,79]. A proportion of this new woodland is planned to be close to urban areas to facilitate people's access and enjoyment of the outdoors [75], see §5 of this paper.

In addition to increased forest cover, there is a drive to improve woodland quality in terms of biodiversity potential and aesthetics for recreational purposes [80,81]. Existing semi-natural broadleaf woodlands, which are important for biodiversity and conservation, are often highly fragmented and embedded in a landscape of agriculture, commercial coniferous plantations and moorland [80,82]. To aid biodiversity, targets have been set to increase the area of semi-natural mixed/broadleaf woodland and to reduce fragmentation by developing ecologically functional forest habitat networks that facilitate the colonization and movement of animals and plants [77,83]. Re-wilding initiatives also aim to restore habitats, and native broadleaf woodland regeneration can form part of these projects. As a consequence, semi-natural mixed/broadleaf woodland is now the most commonly planted woodland type. These changes in woodland cover, type and connectivity are predicted to result in changes to host communities, tick populations and Lyme borreliosis risk.

As well as ecosystem-service benefits from increased woodland, there can be disservices such as increased numbers of pests, such as insects, which are damaging for agriculture or disease-transmitting vectors [78]. Although *I. ricinus* ticks can be found in meadows, open hillside and heather moorland, the highest densities of *I. ricinus* are typically found in woodland. This is due to more favourable abiotic conditions that promote increased tick activity and survival, and due to increased densities of hosts [12,13,27,48,84]. Desiccation is a significant risk to *I. ricinus* survival as the majority of the life cycle is spent in the environment. The saturation deficit, a product of temperature and humidity and a measure of the drying power of the environment, affects the likelihood of *I. ricinus* host seeking or questing [85]. Decaying leaf litter, ground vegetation and canopy cover in woodlands tend to provide more favourable microclimatic conditions than surrounding grassland, moorland or farmland, with reduced saturation deficit allowing more frequent and prolonged questing activity [13]. Importantly, there are also generally higher densities of hosts (especially birds, rodents and roe deer) in woodlands in comparison to moorland and grassland habitats, which increases the probability of ticks obtaining a blood meal.

Based on reported habitat-specific densities, reservoir host densities for *B. burgdorferi* s.l. such as rodents, shrews and birds are predicted to increase as open habitats are converted into woodland [31–33]. This could lead to an increased prevalence of *B. burgdorferi* s.l. in questing nymphs, and, unless tick control measures are put in place, increased densities of infected ticks and an increased risk of Lyme borreliosis. Indeed, a study from central France found a higher prevalence of *B. burgdorferi* s.l. in *I. ricinus* collected from woodland habitat compared to adjacent pasture [86]. Similarly, a study from Scotland found densities of infected *I. ricinus* nymphs (a measure of the risk of Lyme borreliosis) to be five times higher in woodland compared to adjacent open habitats [34].

There is some evidence that the type of woodland may be important for the risk of Lyme borreliosis. In New York State a positive association between *B. burgdorferi* s.s. infection in small mammals and woodlands with lower canopy height and increased amounts of denser ground vegetation was detected [87]. An association between a higher prevalence of *B. burgdorferi* s.l. in questing ticks from semi-natural mixed/broadleaved woodlands compared to coniferous plantation was found in surveys from northern England and Scotland [19,20]. This was suggested to be due to higher densities of bird and rodent transmission hosts in semi-natural woodlands compared to plantations. However, a third study from Scotland did not find a difference in prevalence of *B. burgdorferi* s.l. or risk of Lyme borreliosis in semi-natural mixed/broadleaf woodland compared to coniferous plantations, indicating the difficulty in generalizing between broad habitat types [66]. The distribution of woodlands across the landscape, including levels of connectivity, fragmentation and patch size, will affect the movement of animals, tick abundance and persistence and risk from tick-borne pathogens. Increasing woodland fragmentation has been found by some researchers to be associated with increased *B. burgdorferi* s.l. prevalence and risk [88]. This may be caused by ‘edge effects’ and an increased proportion of suitable ecotonal habitat leading to higher densities of small mammals [86,89], increased densities of roe deer, which can maintain tick populations, and suitable humid microclimatic conditions, which are important for tick survival. Similar ecotonal habitat is found in woodland rides (linear non-wooded herbaceous habitat alongside tracks in woodlands) [13]. Management of woodland rides to maximize biodiversity by reducing scrub encroachment and encouraging wide ‘sunny’ rides increases browsing by deer and provides habitat suitable for small mammal reservoir hosts. This combination of ecological factors could result in increased densities of infected ticks [13]. Therefore, the creation of additional small woodlands and management for biodiversity may lead to higher densities of infected ticks and an increased risk of Lyme borreliosis particularly within ecotonal habitat at woodland edges and along woodland rides. Incorporation of tick-targeted management such as seasonal mowing and management of mulch/mat could be considered to mitigate this increase [13].

The spatial arrangement and connectivity of woodland patches is likely to affect the risk of Lyme borreliosis due to effects on host movement patterns. Isolated and fragmented small forest patches, such as those surrounded by agricultural land in France, or wooded islands within a large water body in Scotland, had a lower risk of Lyme borreliosis compared to nearby continuous forest [90,91]. This is in contrast to reports of increased Lyme borreliosis risk with increased forest fragmentation and smaller forest patch size in North America [88]. In North America, increased Lyme borreliosis risk was associated with decreased mammal-species diversity in smaller forest patches and increased densities of a competent reservoir host, the white footed mouse (*Peromyscus leucopus*). Inclusion of the surrounding landscape in a study of the effect of habitat fragmentation on *I. ricinus* abundance in northern Spain found that ‘stepping stone’ patches of habitat that facilitate host movement had the highest tick density, while patches of suitable but isolated habitat away from main movement networks had lower tick density [92]. Landscape structure and fragmentation of woodland may also affect the genospecies composition of *B. burgdorferi* s.l. This is of public health significance as different genospecies can have different clinical presentations in humans [93,94]. In a study of small wooded islands in a large lake in Scotland, only bird-associated and generalist genospecies were present in fragmented woodland habitat on islands, while both mammal- and bird-associated genospecies were present nearby on the mainland [91]. Lack of persistence of mammal-associated *B. afzelii* on islands may be associated with rodent populations falling below a critical community size during troughs in population cycles, and restricted host movements between islands [91,95].

Natural regeneration of woodlands is often limited by grazing herbivores, particularly deer [77]. It is therefore generally essential to cull deer or exclude them by fencing in order to protect new saplings for the creation of woodlands, as described in §2 of this paper (figure 2). If large herbivores are successfully excluded from regenerating woodlands, this should mitigate the otherwise predicted increase in tick abundance and the risk of Lyme borreliosis. Increasing woodland cover without long-term strategies for deer management could lead to an increased usage of woodlands by deer, increased tick populations and elevated Lyme borreliosis risk.

## 4. Invasive-species management

**Figure 3.**
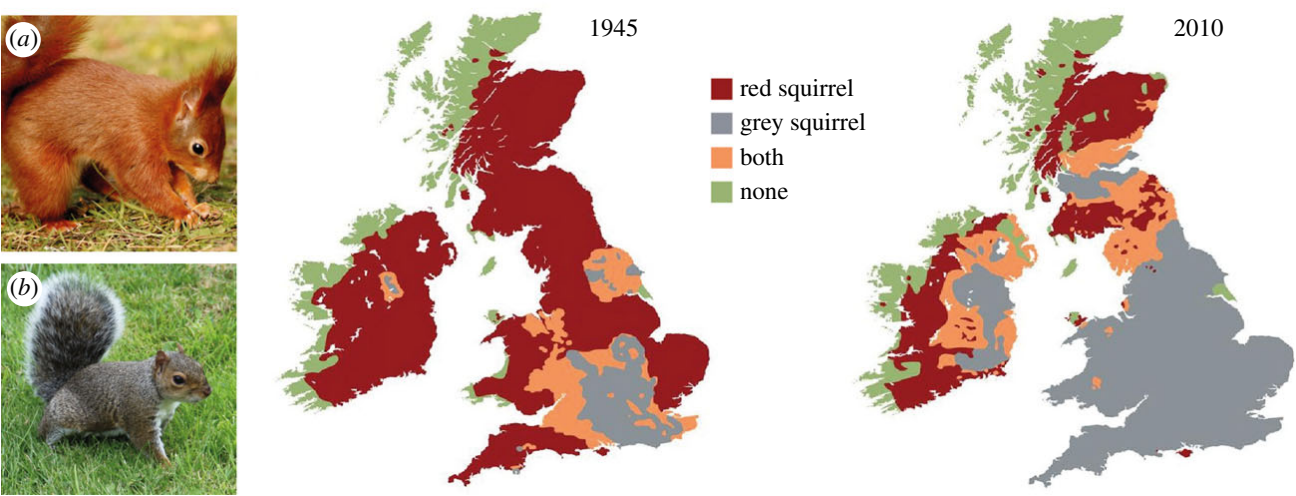
Maps of red (*Sciurus vulgaris*) (*a*) and grey squirrel (*Sciurus carolinensis*) (*b*) distribution in the United Kingdom in 1945 and 2010. (Distribution maps © Red Squirrel Survival Trust, red squirrel photograph © Steve Ransome, grey squirrel photograph © Aileen Adam).

Introduced and invasive species are a widespread challenge in natural-resource management and can affect biodiversity, ecosystem function and human health [96]. Negative effects of these species on native communities can involve predation, direct competition and the introduction of novel parasites [2,97]. In addition, introduced species can act as hosts for endemic pathogens and change the transmission dynamics and infection risk for native species and humans [2]. For example, Siberian chipmunks (*Tamias sibiricus barberi*) introduced to France have been found to be infected with multiple species of *B. burgdorferi* s.l., to harbour more ticks and to contribute more to the local risk of Lyme borreliosis than native rodents [98–100]. The North American grey squirrel (*Sciurus carolinensis*) is an invasive species of major conservation concern in the UK. Following introduction to a small number of sites in the late 19th and early 20th century, this species has invaded large parts of the UK, and now has an estimated population of over two million [31,101] (figure 3). Introduced grey squirrels in the UK thrive in urban parks and gardens as well as rural woodlands [101]. Their introduction has coincided with the decline of the formerly widespread native red squirrel (*S. vulgaris*; see figure 3)—probably the result of direct and/or apparent competition; grey squirrels are asymptomatic carriers of the squirrelpox virus, which is highly pathogenic to red squirrels [97,102–104]. As a consequence, plans for red squirrel conservation rely on halting the spread and reducing the range of grey squirrels through culling. These control efforts are concentrated on areas where the red squirrel is still present, such as large parts of Scotland (figure 3), and have led to recovery of red squirrels in some areas [35]. Targeted culls are also carried out in many other parts of the UK to protect to woodlands, since grey squirrels cause significant damage to woodlands by stripping bark from young trees [101]. This can affect broadleaf/semi-natural woodlands planted for conservation and biodiversity as well as economically important conifer plantations.

The effect of removal of grey squirrels on Lyme borreliosis risk will depend on their relative contribution to pathogen transmission and the effect on the host community competence following their removal, including changes in other reservoir host species densities. Grey squirrels in the UK have been found to carry relatively high numbers of immature *I. ricinus*, they are commonly infected with *B. burgdorferi s.l.*, and infected individuals are able to transmit the pathogen to feeding larvae [105–107]. In Scotland, grey squirrels have been found to be infected with all genospecies found in questing ticks, and most commonly with *B. garinii*, a genospecies normally associated with bird hosts [107]. In addition, this study found that *B. burgdorferi* s.l. prevalence in grey squirrels rose and fell seasonally with changes in tick activity, indicating that infection in grey squirrels might be relatively short-lived [107]. Overall, infection patterns in invasive grey squirrels suggest that the species might be a spill-over rather than a maintenance host for *B. burgdorferi* s.l. [107].

While our current understanding of the role of grey squirrels in Lyme borreliosis epidemiology is still incomplete, the information available indicates that the removal of grey squirrels by itself could result in a reduction in Lyme borreliosis risk, as they host immature ticks and can transmit locally circulating strains of *B. burgdorferi* s.l. [105–107]. However, further studies are needed in particular focusing on the survival of ticks fed on grey squirrels compared to native hosts, and to measure the transmission competence of grey squirrels for different *B. burgdorferi* genospecies. In the long term, effects of grey squirrel removal will depend on the response of the host community, particularly other reservoir hosts, including red squirrels, small mammals and songbirds. Several studies have associated red squirrels with *B. burgdorferi* s.s., and it is possible that the prevalence of this genospecies could rise in local areas where populations of red squirrels increase [108–110]. There is some evidence that grey squirrels may affect populations of songbirds and small mammals [111], which are competent hosts for *B. garinii* and *B. afzelii*, respectively, so the transmission dynamics of these genospecies could also change. Dedicated studies to determine the effects of grey squirrel removal on tick abundance and *B. burgdorferi* s.l. prevalence are clearly needed to better understand and predict the consequences of grey squirrel culling management decisions. As *B. burgdorferi* s.l. strains detected in grey squirrels may reflect the locally circulating strains in questing ticks, this species may be useful as a sentinel for public health surveillance purposes [107]. Studies comparing strains in grey squirrels and in ticks at a finer scale within local woodland areas would be of great interest to investigate their usefulness as sentinels further.

While grey squirrels represent the most obvious example for a potential link between invasive species management and the risk of Lyme borreliosis in the UK, such a link might also exist for other non-native species. For some, the effect could be negligible. Sika (*Cervus nippon*) and fallow deer (*Dama dama*) for example are thought to be equally non-competent to transmit *B. burgdorferi* s.l. and occur at similar densities as native deer species, so their role in Lyme borreliosis epidemiology is likely to be similar. Wild boars (*Sus scrofa*) have been accidentally re-introduced to a few places in England and can host *I. ricinus* [112], though their role as reservoir hosts is uncertain. In contrast, in another introduced species the ring-necked pheasant (*Phasianus colchicus*), high tick burdens, *B. burgdorferi* s.l. infection and transmission of *B. garinii* and *B. valaisiana* to feeding immature *I. ricinus* have been demonstrated [22,25,106,113]. Over 20 million pheasants are released for hunting each year in the UK [114]. This raises questions about the effect of management of host densities on the risk of Lyme borreliosis, but with pheasants being a popular and economically important game bird rather than a designated pest species as for grey squirrels.

## 5. Urban greening

There are several drivers for urban greening, including climate change mitigation to keep cities cooler, and peri-urban woodland regeneration and restoration projects for human well-being and biodiversity. Encroachment of towns and cities into woodland areas and increasing urban and peri-urban greenspace also increase the likelihood of human–tick contact and exposure to tick-borne pathogens. A recent review of the likely impacts of climate change on vector-borne disease in the UK has raised concerns over the possible indirect effects on vector-borne disease systems via climate change mitigation [9]. In line with these predictions, a tick surveillance scheme run by Public Health England has found increased reports of ticks acquired in urban areas (Hansford K, Medlock J. 2016 personal communication). This could suggest that like other urban areas in Europe, similar UK habitats may also be suitable for ticks and may therefore pose a hazard to humans from tick-borne diseases.

For *I. ricinus* to survive in urban areas, it needs the same ecological and environmental requirements that it has in the countryside; having access to suitable vegetation and animal hosts. The requirement for low saturation deficit (including a high relative humidity of over 80%) to reduce desiccation-associated mortality [85] restricts *I. ricinus* to urban parks, forest patches and gardens. Urban areas can provide a mosaic of ecotonal habitats, with areas of woodland, hedges, grasslands managed as meadows for flowers and insects, and parks all in close proximity. In some cases, expansion of towns has brought ancient woodland, and more traditional tick habitat, closer to the urban environment, with some cities engulfing woodland within its limits. Urban areas are home to many wildlife tick hosts including increasing numbers of urban deer and red foxes (*Vulpes vulpes*) [115,116], but also dogs and cats which can provide an abundant host for the nymphal and adult tick stages [36]. The proportion of ticks feeding on dogs and cats which are able to return to habitat that could support their development is unknown; however, it is likely that some engorged ticks will find suitable habitat to develop a subsequent generation. Dogs and cats may also carry ticks into human homes and increase human–tick exposure. Higher temperatures in urban areas compared to surrounding rural areas may also affect tick development rates [117,118] and tick–host interactions.

Like many organisms, ticks also benefit from habitat connectivity, and urban areas with well-connected habitats via green corridors would probably support more ticks. Also, urban greenspace on the margins of towns and cities, with direct connectivity to the countryside, may also be significantly more suitable for tick survival than fragmented habitats. These connected spaces are also used by members of the public (walking or walking dogs) to navigate through urban areas, providing opportunities for exposure to ticks and potentially the movement of ticks between fragmented habitat on canine hosts. Urban tick densities are likely to be lower than those in the countryside, due to lower host density, but human exposure is likely to be much higher. The latter factor is difficult to quantify, but it is conceivable that low tick densities in a publicly accessible site will present a greater risk to human health than high tick densities in a remote woodland [37,119–121]. Behavioural research directed towards understanding the probabilities of human–tick contact in different environments is needed; human disease risk cannot be estimated with any accuracy with data relating to the environmental risk alone (i.e. density of infected nymphs).

The first report indicating a risk of Lyme borreliosis in urban green space in the UK identified a clinical case of *B. burgdorferi* s.l. infection in a dog that had visited Richmond and Bushy Park in London in 1988, and testing of questing ticks from the parks found a prevalence of *B. burgdorferi* s.l. of 8% [122]. Serological evidence of *B. burgdorferi* s.l. exposure was later detected in park workers [123]. Many further studies on *B. burgdorferi* s.l. prevalence in ticks have included Richmond Park as a site of interest [21,124–126], but this does not represent a typical urban area and its large population of red deer contribute significantly to tick survival. More recent research in cities in Southern England have investigated tick density and the prevalence of *B. burgdorferi* s.l. in more typical urban greenspace such as small parks, vegetated pathways, housing estates bordering woodland, urban grasslands managed as meadows, as well as woodlands within urban settings. Higher tick densities were found in urban woodlands or localities (e.g. parks, grasslands) adjacent to woodland, particularly on the urban fringe. Seasonal variation in *B. burgdorferi* s.l. prevalence and tick densities was found and high *B. burgdorferi* s.l. prevalence rates of up to 30% were detected in some of these habitats [37] compared to a mean of 7.5% in woodland habitat from outside urban areas in England [20]. Early findings suggest that the presence and prevalence of *B. burgdorferi* s.l. infected ticks across tick-infested habitats are heterogeneous, lending strength to the importance of connectivity in determining *B. burgdorferi* s.l. prevalence as well as tick density. Differences in habitat connectivity could impact the ability for larger animals (e.g. deer) to access urban habitats, thus increasing tick densities, but possibly reducing *B. burgdorferi* s.l. prevalence. There is also evidence from across Europe that urban habitats support ticks and associated tick-borne pathogens [127–129], with various studies comparing tick abundance and *B. burgdorferi* s.l. prevalence in both rural and urban areas [130–135], and others looking at variation across different urban green spaces [119,127,132,135–137].

For increasing parts of southern England at least, ticks appear to be emerging as an urban issue (Medlock J, Hansford K. 2016 personal communication), and owing to the complex ecology of *B. burgdorferi* s.l., the hazard of Lyme borreliosis in such areas might be comparable to more typical, rural habitats. Similar mitigation strategies can be employed in both rural and urban areas to help reduce expose to ticks but the challenge now is how we use these control measures alongside managing greenspace for nature and the health and well-being of the public.

## 6. Conclusion

There are many positive effects of conservation and biodiversity management, including benefits to human well-being from spending time in nature [138]. As well as these benefits, any strategy that increases biodiversity may also increase the abundance of some organisms considered to be pests, such as vectors, and could result in a risk to public health. This review synthesizes current knowledge on the effects of conservation management of landscapes and host communities on the risk of Lyme borreliosis in the UK. This is useful to identify knowledge gaps and future research directions, and similar approaches could be made with other infectious-disease systems. Each of the conservation management actions, including deer management, woodland regeneration, invasive species management and urban greening, is predicted to result in changes to host communities and movements, tick abundance and the risk of Lyme borreliosis (table 1). Understanding and predicting the effects of particular management changes on the risk posed by a vector-borne pathogen can be complex [139]. For example, from this review, removal of a large herbivore such as deer can result in cascading effects on vegetation, small mammals and vector populations resulting in altered pathogen transmission [12].

There is significant uncertainty in the effects of some of these types of management considered in this review on Lyme borreliosis risk due to a lack of empirical studies. Land use management actions are essentially large-scale ecological experiments, which are otherwise rare and difficult to conduct. As such they provide unique opportunities to gain mechanistic insights about ecological interactions relevant to disease transmission [140]. Research studies to answer knowledge gaps outlined in each of the main sections could be designed as part of existing or new conservation projects in order to answer these questions. For example, where biodiversity monitoring is conducted to measure the success of conservation projects such as woodland regeneration or restoration, tick surveys and pathogen testing could be incorporated as part of these studies.

As the cycles of many vector-borne pathogens occur in nature, interdisciplinary collaboration between veterinary and human public health scientists with stakeholders in conservation and forest management is needed to assess and minimize these environmental hazards [10,141]. Although conservation management decisions will necessarily consider many factors, we suggest that inclusion of vector-borne pathogen dynamics and mitigation should be part of environmental impact assessments [142]. It will be important for habitat creation projects to demonstrate that in addition to measuring increases in biodiversity, such projects do not create pest and vector issues. Indeed such a lack of assessment and monitoring has blighted a number of wetland creation projects in relation to mosquito risk [143]. Ideally, the review of information and assessment of whether the risk of a vector-borne pathogen will increase or decrease with a conservation management action would be part of an interdisciplinary framework, with involvement of stakeholders in medical and veterinary public health, environmental management and biodiversity enhancement to provide advice to government [10,141].

In line with efforts in relation to managing habitats for mosquitoes [142], further research on how conservation management impacts Lyme borreliosis risk would inform best-practice guidelines for practitioners in how to manage woodlands accessible by the public in order to minimize the exposure of people to infected ticks. This could include guidelines on environmental management to minimize the hazard from ticks, for example by managing the height of ground vegetation close to paths [13]. Another important measure would be to provide appropriate risk messaging about ticks and tick-borne diseases, with surveys to assess the effectiveness of these measures [79].
